# Reticulon Protein-1C: A New Hope in the Treatment of Different Neuronal Diseases

**DOI:** 10.1155/2012/651805

**Published:** 2012-05-24

**Authors:** Federica Di Sano, Mauro Piacentini

**Affiliations:** ^1^Department of Biology, University of Rome “Tor Vergata”, Via della Ricerca Scientifica, 00133 Rome, Italy; ^2^National Institute for Infectious Diseases IRCCS “L. Spallanzani”, Via Portuense, 00149 Rome, Italy

## Abstract

Reticulons (RTNs) are a group of membrane proteins localized on the ER and known to regulate ER structure and functions. Several studies have suggested that RTNs are involved in different important cellular functions such as changes in calcium homeostasis, ER-stress-mediated cell death, and autophagy. RTNs have been demonstrated to exert a cancer specific proapoptotic function via the interaction or the modulation of specific proteins. Reticulons have also been implicated in different signaling pathways which are at the basis of the pathogenesis of several neurodegenerative diseases. In this paper we discuss the accumulating evidence identifying RTN-1C protein as a promising target in the treatment of different pathologies such as cancer or neurodegenerative disorders.

## 1. Introduction

Neuronal death occurs by necrosis or apoptosis, which differ morphologically and biochemically. Necrosis is the result of extreme perturbation of the cellular environment, as occurs in ischemic insults or trauma. In contrast apoptosis is dependent on intracellular pathways which lead to cellular commitment to a defined series of steps leading to cell suicide. Apoptosis is an important mechanism in normal cell turnover and in growth and development, as well as in aging. Alteration of this machinery results in the evolution of cancer and autoimmune or degenerative diseases. The process of neuronal apoptosis involves two principal pathways that converge on activation of caspases: the cell surface death receptor pathway and the mitochondrial pathway [1]. However, a role in the initiation of neuronal apoptotic cell death by other organelles, including endoplasmic reticulum (ER), is now well established [2].

Disruption of ER homeostasis interferes with protein folding and leads to the accumulation of unfolded and misfolded proteins in the ER lumen. This condition, designated “ER stress,” can be triggered by stimuli that perturb ER function, including depletion of Ca^2+^ stores, reduction of disulphide bonds, overexpression of certain proteins, and nutrient/glucose deprivation [3]. To maintain homeostasis, the ER mounts an unfolded protein response (UPR), as a self-protective mechanism, which results in transcriptional induction of UPR genes, translational attenuation of global protein synthesis, and ER-associated protein degradation (ERAD) [3]. However, if these adaptive responses are not sufficient to relieve the ER stress, the cell dies through apoptosis [3]. Interestingly, prolonged ER stress is at the basis of the pathogenesis of several neuronal disorders [4, 5]. In fact, recent studies have reported that many human disorders have their origin from dysfunctions in the endoplasmic reticulum and that regulation of some important UPR mediators may be potential targets for modulating ER stress response [6].

For example, ER-stress-dependent apoptosis is important in ischemia-reperfusion injury and in neurodegenerative diseases (such as Alzheimer's and Parkinson's), where abnormalities have been identified in protein folding or secretion in the Golgi-ER compartment [4].

In the last few years a new family of proteins, reticulons (RTN), primarily localized on the ER membrane, has attracted particular interest due to their implication in different cellular processes [7]. They play important role in bending and shaping the ER membrane, in trafficking of material from the ER to the Golgi apparatus, and in apoptosis [8–10]. In particular, studies from different groups have indicated that RTN proteins show a proapoptotic function mediated by the induction of endoplasmic reticulum stress [11, 12] and also act as key regulators of ER morphology and nuclear envelope formation [13].

The mammalian reticulon family of proteins consists of four members (RTN1–RTN4); the specific functions for most of them are presently poorly understood although RTN4 (also called Nogo) has been widely demonstrated to be an inhibitor of axonal extension and neurite outgrowth [14]. RTN genes are expressed as multiple N-terminal isoforms that are generated by the use of different promoters or alternative splicing events [15]. Their C-terminal reticulon domain is evolutionarily conserved while the specificity of the N-terminal region contributes to their interaction with a vast array of proteins [16–19]. Although the reticulon genes are expressed in many tissues and are conserved in different phyla, suggesting a basic function in cell physiology and a universal role in the eukaryotic system, some of them (RTN-1A, RTN-1C, Nogo-A and RTN-2) are preferentially expressed in nervous tissues [20].

Reticulons were originally identified as markers for carcinomas with neuroendocrine characteristics [21]. In particular, the reticulon family gene 1 (RTN-1) was characterized by antibodies that stained a subset of neuroendocrine tissues and neoplasms [21] and was formerly called neuroendocrine-specific protein.

There is no doubt that cellular homeostasis of RTNs is important for normal cellular function. In particular, the pattern of RTNs localization in the ER, Golgi, and plasma membrane strongly suggests the existence of trafficking functions in the secretory compartment. In line with this finding it has been demonstrated that reticulons interact with several proteins regulating endo- and exocytosis processes [22].

They also have been identified as proteins showing a peculiar and almost exclusive localization to the tubular ER [23]. Moreover they contain a domain of ~200 amino acids (RHD), including two hydrophobic segments of unusual length (30–35 residues), which are thought to form a hairpin in the membrane of endoplasmic reticulum [23]. These structural properties, together with reticulons' ability to oligomerize, allow these proteins to generate and/or stabilize tubules *in vitro* and to play a key role in the mechanism by which the tubular network of the endoplasmic reticulum is generated and maintained [23]. It has been also demonstrated that reticulon proteins are deeply involved in nuclear envelope formation during mitosis in metazoans by a chromatin-mediated reorganization of the tubular ER [13]. In fact, it has been suggested that the levels of reticulons directly affect the balance between tubules and sheets in the ER and contribute to nuclear envelope formation [13].

On the other hand, more recent studies have expanded the biological functions of RTNs in several neuronal disorders [24–26], including cancer of cells of neuronal origin or neurodegeneration pathologies such as Alzheimer's disease and amyotrophic lateral sclerosis (ASL) [27]. It has been also demonstrated that reticulons participate in and regulate the pathogenesis of other diseases such as atherosclerosis [28].

## 2. RTN-1C and Brain Tumors

Cancer is characterized by an imbalance between cell division and cell death, caused by homeostatic changes occurring during the transformation process, the consequence of which results in dysregulation of apoptosis. Different therapeutic approaches have been used to counteract tumors growth and induce apoptosis, largely based on the induction of p53-dependent DNA damage. However, since more than 50% of tumors are defective in p53-family transcriptional activity, with mutations resulting in abrogated protein function and genetic instability, many tumors are insensitive to these treatments, leaving an acute need for novel therapies able to induce apoptosis independently of p53. This need is apparent even in tumors lacking in (or with rare occurrence of) p53 mutations, such as neuroectodermal tumors, a family of tumors notoriously resistant to apoptosis due to defective activation of apoptotic signalling pathways mediating either death receptor ligation or DNA damage [29–31]. Furthermore, these tumors remain resistant to p53-dependent damage despite p53 status being rarely mutated [32–34] as reported for many other types of cancer [35]. The poor response to chemotherapy thus results in poor survival rates, leaving us in acute need of novel therapeutic strategies. It has been suggested that inducing apoptosis via ER stress may represent a novel way to kill cancer cells that are resistant to apoptosis mediated by death-receptor ligation or DNA damage [36].

Reticulons are expressed in most neuroendocrine tumors; they are considered to be highly sensitive and specific markers of neuroendocrine differentiation for use in the diagnosis. In this context it is interesting that reticulons can exert a cancer-specific proapoptotic function. Such evidence is particularly pertinent for the RTN-1C family member. The first experimental results in this regard detected RTN-1C capability of interacting with glucosylceramide synthase (GCS) (Figure 2), a key enzyme in the biosynthesis of glycosphingolipids, and implicated in many biological phenomena, including multidrug resistance (MDR) [37]. Various studies have demonstrated a direct correlation between the development of MDR and increased levels of glucosylceramide [37, 38], with GCS being suggested as a candidate target for cancer therapy.

GCS inhibition, by both antisense and the specific inhibitor (D-threo)-1-phenyl-2-decanoylamino-3-morpholino-1-propanol (PDMP), results in a drastic decrease of apoptosis induced by the p53-independent chemotherapeutic agent *N*-(4-hydroxyphenyl)retinamide (fenretinide) in neuroepithelioma cells [39]. Significantly it has been demonstrated that RTN-1C not only interacts with GCS at Golgi/ER interface but also modulates its catalytic activity *in situ* and affects the apoptotic response to fenretinide-induced apoptosis via a p-53 independent mechanism [40].

Thus GCS role in fenretinide-induced apoptosis is influenced by RTN-1C, which provides a functional link between Golgi and ER in this response. These results confirmed the possibility of developing a targeted therapy for cancer using a combination of p53-dependent and p53-independent pathways. In line with this, we demonstrated that RTN-1C regulates in a mutually exclusive way ER stress versus DNA-damage-induced cell death [11]. In fact, the increase of RTN-1C protein levels *per se* results in endoplasmic reticulum stress-induced cell death, mediated by an increase of cytosolic Ca^2+^. This increase also significantly sensitizes cells to different endoplasmic reticulum stress inducers [11]. In line with these findings, the reduction of RTN-1C, by means of antisense DNA expression, reduced the response to ER stressors [11]. In the presence of high RTN-1C levels, genotoxic drugs become ineffective as a consequence of the cytoplasm translocation of p53 protein; conversely the silencing of endogenous RTN-1C boots the effectiveness of genotoxic drugs [11]. These data indicate that RTN-1C is able to modulate the cellular sensitivity to different apoptotic pathways and so it represents a promising molecular target for new drug development.

Other interesting data provided by experiments demonstrated that RTN-1C expression is directly correlated to calreticulin (CRT) exposure on the plasma membrane; this is through a mechanism mediated by the reduction of endoplasmic reticulum Ca^2+^ [41]. Such an event has been proved to be important for the activation of anticancer immune response and cell death. In fact, CRT exposure has been correlated to immunogenic cell death after anthracycline and *γ*-radiation in mouse models and has the potential for rendering conventional chemotherapies immunogenic [42, 43].

**Figure 1:**
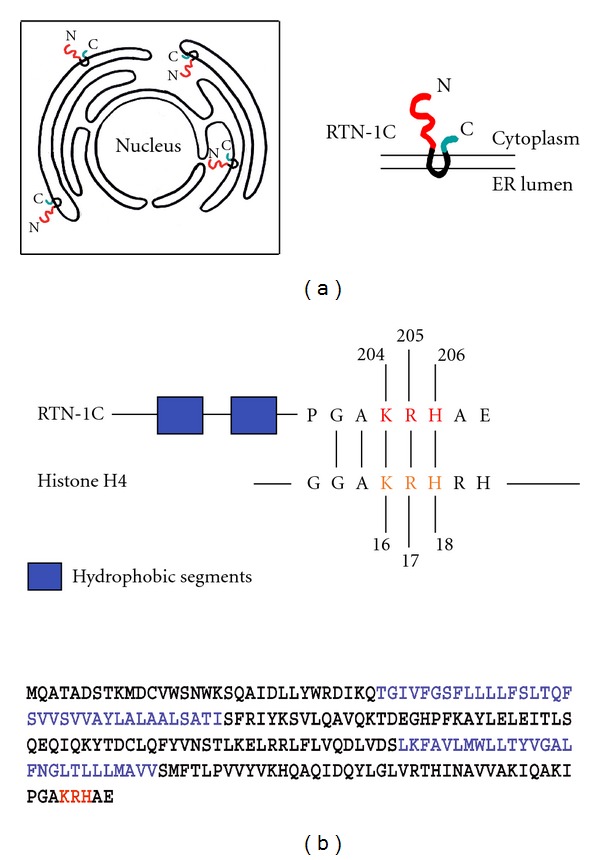
(a) Scheme showing the reticulons distribution on endoplasmic reticulum and the physical connection between nuclear envelope and ER membrane. (b) Schematic diagram of RTN-1C and histone H4 proteins showing the shared GAKRH motif. The blue aminoacids indicate the two hydrophobic segments of RTN-1C protein. The red aminoacids indicate the three positive charges in the H4 consensus motif. RTN-1C is acetylated on Lys 204.

More recently it has been reported that the synthetic peptide corresponding to the C-terminal region (aa 179–208) of the human RTN-1C is able to bind DNA. The C-terminus of RTN-1C is characterized by the presence of an H4 histone consensus motif [PS000047 HISTONE_H4] (GAKRH) [44] (Figure 1), the lysine present in this consensus sequence being one of the four residues that can be acetylated and modulate the H4 histone interaction with DNA [45].

Lysine acetylation is a reversible and highly regulated posttranslational modification, initially discovered on histones, and known to regulate diverse protein properties including DNA-protein interaction, subcellular localization, transcriptional activity and protein stability [46]. Acetylation modifies the lysine residues of both histone and nonhistone target proteins, such as p53, and is now recognized as an important regulatory step in gene transcription. Moreover lysine acetylation and its regulatory enzymes (acetyltransferases, HAT and deacetylases, HDAC) have been intimately associated with many different diseases, such as cancer and neurodegenerative disorders [47, 48] and with different cellular functions including stress response and apoptosis [49]. In this regard epigenetic regulation of gene transcription has recently attracted wide interest in the field of cancer especially because several genes implicated in oncogenesis are regulated by acetylation and deacetylation [50]. Modification of proteins by histone acetyltransferases or histone deacetylases plays an important role in the control of gene expression while their dysregulation has been linked to malignant transformation and other diseases [48]. Histone deacetylases are recognized as one of the most promising targets for cancer treatment, many HDACs inhibitors currently undergoing clinical trials [51, 52]. These enzymes are nowadays considered among the best potential targets for antineoplastic therapy [51]. HDACs are overexpressed in many cancer cells, and the death-inducing capability of different HDAC inhibitors correlates with their inhibitory potency. Interestingly these compounds have been demonstrated to induce apoptosis mediated by caspase activation as well as caspase-independent autophagic cell death [53]. Induction of two modes of programmed cell death by HDAC inhibitors indicates that these drugs might be particularly valuable when treating cancers with apoptotic defects.

In particular, inhibition of HDACs results in growth arrest and apoptosis of cancer cells while their deregulation has been linked to autophagy induction [54], suggesting that the epigenetic regulation of gene expression may be a fertile area for the development of anti-cancer strategies.

**Figure 2:**
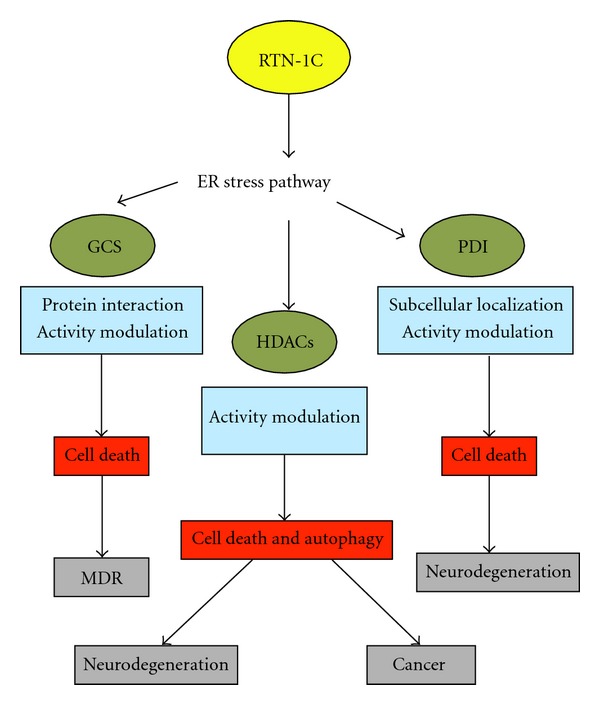
Schematic representation of the different RTN-1C-induced signaling pathways. Modulation of RTN-1C expression triggers the ER stress pathway and the regulation of different proteins (green ovals) at different levels (blue boxes). These events affect cellular processes (red boxes) which are at the basis of several human pathological settings (grey boxes).

As previously reported, an H4 histone consensus motif has been identified in the C-terminal domain of RTN-1C. All the three positively charged amino acids (KRH) in the H4 consensus are essential for RTN-1C-mediated apoptosis. The function of this reticulon protein has been shown to be modulated by posttranslational acetylation on lysine 204. Worth noting is a direct correlation between RTN-1C acetylation and HDACs activities; in fact the reticulon protein is able to negatively modulate their enzymatic activity (Figure 2). Moreover, RTN-1C protein is able to bind to DNA, this interaction being regulated by the acetylation process [45].

Considering the critical role of lysine acetylation in regulating diverse cellular functions, such as cancer development and human brain disorders [48, 51], HDAC substrates could represent candidate proteins relevant to human disease and therapeutic targets for drug design. In this context RTN-1C not only represents an important candidate for the development of new epigenetic therapeutic strategies, but also a novel protein with affinity for DNA, which could regulate the interaction between the ER/nuclear envelope membranes and chromatin.

## 3. RTN-1C and Autophagy

Another interesting connection between RTN-1C and cancer development may be the reticulon possible involvement in autophagy induction. Autophagic cell death is another important physiological cell death process, involved in development and stress responses. Furthermore, like apoptosis, autophagic cell death is involved in tumorigenesis, even if currently there is no cancer therapeutic approaches that specifically target the autophagic cell death machine.

It is now well accepted that there is a complex interplay between apoptosis and autophagy and that the molecular regulators are interconnected; numerous death stimuli are capable of activating either pathway and both pathways share several genes that are critical for their respective execution [55]. In particular, several reports have indicated a functional link between ER stress signaling pathways and autophagy. This is highlighted firstly by the possibility that endoplasmic reticulum stress response may lead to the induction of autophagy, and secondly by the fact that deregulation of this process causes several disorders characterized by the accumulation of toxic proteins in the ER [56]. The cross-talk between apoptosis and autophagy is critical and represents a key factor in the outcome of death-related pathologies such as cancer, its development and treatment. In particular, accumulating evidence suggests that ER stress is linked to autophagy [57, 58], where inhibition of the apoptosis pathway induces activation of the autophagy programme and vice versa [55, 59]. Moreover it has been shown that proper function of ER is required for autophagosome formation; when the ER senses the accumulation of unfolded misfolded protein, it can signal the induction of autophagy to overcome the resulting stress [60]. Finally, the possibility that autophagy could act as degradation system for unfolded protein accumulated in the ER in addition to ERAD has been demonstrated [61]. Interestingly a role for autophagy in the prion disease process has recently been suggested. In this regard it has been reported that cytoplasmic prion aggregates lead to endoplasmic reticulum stress activation of reticulon 3, impairment of ubiquitin-proteasome system, induction of autophagy, and apoptosis [62]. In this context it seems that RTN3 negatively regulates autophagy.

It has been also suggested that inducing apoptosis via ER-stress and/or autophagy may represent a novel way to kill chemoresistant cells and a promising approach for enhancing efficiency of cancer chemotherapy [37, 63]. Interesting pilot data (manuscript submitted) show that in neuroblastoma cells the modulation of RTN-1C expression disrupts Ca^2+^ signalling and induces Ca^2+^-dependent autophagy. We can assume that autophagy may act as a prosurvival mechanism in response to ER stress and the accumulation of unfolded or misfolded proteins; thus autophagy machinery is perhaps activated to degrade the UPR proteins. Conversely, when the stress becomes severe and prolonged, these fine regulatory mechanisms are not sufficient and the cell undergoes apoptosis. According to this the upregulation of RTN1-C expression results in ER stress induction, finally leading to apoptotic cell death [11]. These data are a compelling reason to further explore the role of RTN-1C in autophagy induction and to understand to what extent and by which mechanism ER-stressed cells escape autophagy protection and commit apoptosis.

Future studies should define the molecular details dictating the alternative behaviour of the different RTN family members in autophagy regulation.

## 4. RTN-1C and Neurodegeneration

Another exciting frontier of reticulon research is the field of neurodegenerative diseases. It has been widely demonstrated that the ER stress pathway is implicated in various neurodegenerative pathologies [6]. For example UPR activation has been shown to be an early event in the brain of Alzheimer's patients [64]. It has also been observed that motoneurons of amyotrophic lateral sclerosis (ASL) patients are characterized by ER alterations, so suggesting that the UPR response is activated and that ER stress may be involved in the neurodegeneration of these cells in early stages of ASL [65]. In this context, reticulons have been shown to play an important role in the nervous system both in normal and pathological settings, and recent studies have expanded the biological functions of RTNs in several CNS disorders, including Alzheimer's disease (AD) [66–68]. The latter, AD, is a progressive neurodegenerative disorder characterized by cognitive deficits and extensive neuronal loss. Several pathological changes have been described in postmortem brains of AD patients, including beta-amyloid (Abeta) plaques, intracellular neurofibrillary tangles formed by the hyperphosphorylated tau protein, inflammation, and extensive cell death [69]. One of the earliest molecular events in AD patients involves disturbances in calcium homeostasis [70]. Interestingly, it was found that all four human reticulon proteins can modulate BACE1 enzymatic activity [71] thought to contribute to early synaptic loss leading to the initial cognitive decline. In particular, reticulon proteins block access of BACE1 to APP and reduce the cleavage of this protein [72]. Thus, changes in the expression of reticulon proteins in AD brain are likely to affect cellular Abeta and the formation of amyloid plaques in AD [71, 73].

Studies carried out on postmortem brain tissues have revealed changes in reticulon expression in the temporal and frontal cortex of patients with Alzheimer's disease [74]. In particular, it has been demonstrated that the expression of RTN-1C is significantly reduced in the frontal cortex of Alzheimer's disease patients compared to controls [74]. Finally altered RTN expression has been directly correlated to significative effects on cellular trafficking and abnormality in exocytosis or endocytosis; these may represent one of the mechanisms that could lead to neurodegenerative disorders [75, 76].

We have recently shown, by the use of microarray analysis of the whole human genome, that RTN-1C is able to specifically regulate gene expression, modulating transcript clusters implicated in the onset of neurodegenerative disorders [77].

*In vivo* studies have also established that enhanced expression of this reticulon family member in the cerebral cortex results in ER stress, leading to a neurodegenerative process characterized by an abnormal synaptic plasticity at corticostriatal synapses [77].

Another important mechanism involved in neurodegeneration process is the regulation of calcium homeostasis. Dynamic changes in calcium concentration within the ER and alteration of ER calcium homeostasis may be important in regulation of cell function and survival and may trigger various forms of neurodegeneration and/or neuropathy [78, 79]. In fact, disruption of the ER calcium homeostasis triggers ER stress response, which in turn may trigger a cascade of events leading to survival or death of neuronal cells.

Interestingly, RTN-1C modulates the expression of genes which has been demonstrated to be affected in schizophrenia with a common thread related to Ca^2+^ signalling [80]. It has been observed that schizophrenic patients displayed elevated mobilization of Ca^2+^ from intracellular stores in response to receptor stimulation; this suggests that increased cytosolic calcium may be the primary molecular abnormality in this pathology [81]. In keeping with this finding, it is known that the reticulon protein controls cytosolic Ca^2+^ levels by depleting the ER Ca^2+^ stores [11]. Matching these findings, reticulon overexpression has been observed in the cortex of patients affected by different neuronal pathologies including schizophrenia [82]. Furthermore, numerous studies have shown that the ER, whose function is strictly regulated by RTN-1C, plays a number of essential roles in synaptic transmission and plasticity at many central synapses [81]. Conversely, perturbation of ER Ca^2+^homeostasis is critically involved in aberrant forms of synaptic plasticity in mouse models of AD and schizophrenia [4, 83, 84]. In this context, it has been recently established that transgenic mice overexpressing reticulon 3 develop dystrophic neurites with impairment of spatial learning abilities and hippocampal long-term potentiation (LTP), thus resembling AD-like phenotype [85]. RTN-1C involvement in neurodegeneration processes was also investigated by the use of a transgenic RTN-1C mouse model. These mice exhibit maladaptive synaptic plasticity and show disregulation of two plasticity-related genes DARPP32 and NOS2a whose induction/correct modulation is required for normal expression of bidirectional plasticity [86, 87]. Interestingly, Meyer-Lindenberg et al. [88] identified a specific haplotype of DARPP32 gene, associated with the risk for schizophrenia in a family-based association analysis. Altogether, these data highlight an as yet unknown role for RTN-1C for the regulation of higher brain functions associated with motor learning.

Reticulons have recently been implicated in other important neurodegenerative disorders; amyotrophic lateral sclerosis, which is a rapidly progressing fatal neurodegenerative disease, is characterized by the presence of protein inclusion in motor neurons. The induction of ER-stress-mediated apoptosis has proved to be an important event in the pathogenesis of ASL; moreover ER occurs early in the disease and involves the upregulation of protein disulphide isomerase (PDI), an important endoplasmic reticulum chaperone [89]. Endoplasmic reticulum is the primary site for synthesis and folding of secreted and membrane-bound proteins. The accumulation of unfolded and misfolded proteins in ER triggers a wide range of human neurodegenerative disorders. It is now evident that molecules that regulate the ER stress response represent potential candidates as drug targets to tackle these diseases. Protein disulphide isomerase is a chaperone involved in ER stress pathway, its activity being an important cellular defence against protein misfolding. The regulation of PDI activity may be a way of modulating ER stress responses and consequently the balance between apoptosis or survival in stressed cells. An increase of PDI activity could represent a means of counteracting protein inclusion formation typical in neurodegenerative diseases [90].

Several reports suggested that PDI function is particularly important for neuronal cell death because it is able to attenuate the neurotoxicity associated with the accumulation of aggregated proteins which is responsible for neurodegenerative processes [90]. It has been suggested that PDI activity is impaired in ALS. In addition anti-PDI-antibody immunopositive inclusions have been found in neurofibrillary tangles (NFTs) of the brain of Alzheimer's patients [91].

Interestingly, a very recent paper has shown that the reticulon family proteins, and in particular RTN-1C, represent novel regulators of PDI intracellular localization and that this phenomenon could be an important modulating factor in amyotrophic lateral sclerosis [92]. In this context, recent data (manuscript submitted) have demonstrated that the reticulon-1C family member is able to cause a dramatic intracellular PDI redistribution, from a diffuse to a punctate pattern which is not simply the result of ER stress induction. More importantly, RTN1-C significantly increases PDI enzymatic activity by modulating S-nitrosylation reactions (Figure 2). These results are in line with previously known experimental evidence that PDI functional activities (chaperone and isomerase) are regulated by S-nitrosylation processes [90]. Interestingly, it has been reported that inhibition of PDI activity by S-nitrosylation is strongly associated with mutant Cu/Zn superoxide dismutase toxicity in amyotrophic lateral sclerosis disease and that a small molecule mimicking the PDI active site protects against mutant superoxide dismutase 1 inclusion formation [89]. Moreover in models of Parkinson's disease, one of the S-nitrosylated targets found is the PDI [90]. Thus, based on these findings, RTN-1C is a good potential candidate for the modulation of PDI function in ameliorating aggregation and toxicity of mutated proteins.

Another interesting link between neurodegenerative disease and reticulons concerns the latter's previously mentioned involvement in autophagic signaling pathway. Autophagy dysfunction has been extensively described in neurodegenerative conditions linked to protein misfolding and aggregation [93]. Pharmacological induction of autophagy can enhance the clearance of intracytoplasmic aggregate-prone proteins, such as mutant forms of huntingtin, and ameliorate pathology in cell and animal models of neurodegenerative diseases [94].

Recent studies have reported that autophagy is the major degradational pathway following UPR activation in neuronal cells and constitutes a connection between UPR activation and autophagic pathology in AD brain [95]. In line with this assumption, a recent work has reported that RTN3 negatively regulates autophagy blocking the clearance of cyPrP aggregates and thus providing a clue regarding the potential for inducing autophagy for the treatment of prion disease and other neurodegenerative diseases such as Parkinson's disease, Alzheimer's disease, and Huntington's disease.

We have recently found that (manuscript submitted) RTN-1C induces the activation of a Ca^2+^-dependent autophagic pathway which is paralleled by changes in mitochondrial morphology and mitochondrial fission and fusion machinery. RTN-1C-mediated ER stress condition not only triggers alterations in Ca^2+^ homeostasis and mitochondrial dynamics, but is also capable of inducing an autophagic response. In the context of neurodegeneration, autophagy may constitute a prosurvival mechanism in response to ER stress and the accumulation of unfolded or misfolded proteins; reticulon-1C structural ER protein may modulate the neuronal stress at the basis of neurodegenerative pathologies.

Finally very interesting recent work has reported that reticulons are implicated in axonopathy and, in particular, in hereditary spastic paraplegias (HSPs), a group of genetically heterogeneous neurodegenerative conditions. Specifically RTN-2 has been found to be mutated by a complete deletion or a frameshift mutation, producing a truncated protein which is the cause of HSP [96]. These findings are most likely correlated to an abnormal reticulon function in the morphogenesis of the ER resulting in axonal degeneration.

## 5. Conclusion

Reticulons are a family of integral membrane proteins implicated in a variety of important biological functions such as ER morphology and organization, nuclear envelope formation, calcium homeostasis, and cell death. In the last few years many experimental data have expanded the reticulons' functions to a wide array essential for neuronal cell homeostasis Table 1. RTNs deregulations have been implicated in several human diseases such as cancer development and/or neurodegenerative disorders. However, for most of the reticulon proteins the specific role and the biochemical mechanisms at the basis of their biological function are still unknown.

Studies from our group have focused on the reticulon-1C isoform of RTN1 gene which we originally identified as a GCS interacting protein. Considering the role of this enzyme in the mechanism of cancer development and MDR, we have been interested in characterizing the role of RTN-1C in tumour cells of neuroectodermal origin. During the last few years, we have obtained a series of very interesting results in the field of cancer development: these indicate that reticulon-1C is a promising molecular target for novel therapeutic approaches. We next expanded RTN-1C function to the modulation of neurodegeneration processes. We discovered that it participates in different signaling pathways, from ER-stress-induced cell death to autophagy, through the regulation of different enzymes such as HDACs or PDIs; future studies however would need to define the molecular details regulating the RTN-1C biological functions essential for clarifying its involvement in neural cell pathologies.

## Figures and Tables

**Table 1:** Correlation between some RTNs cellular functions and their potential involvement in different human diseases.

Cellular functions	Human diseases
ER homeostasis	Cancer and neurodegenerative disorders (i.e., ALS, Alzheimer's disease)
Calcium homeostasis	Neuronal pathologies (i.e., schizophrenia)
Apoptotic response	Cancer, neurodegenerative disorders (i.e., ALS, Alzheimer's disease Parkinson's disease)
Membrane trafficking	Neurodegenerative disorders (i.e., Parkinson's disease)
Autophagy	Cancer, neurodegenerative disorders (i.e., prion disease, Alzheimer's disease, Huntington's disease)
